# Multidisciplinary Management of Retroperitoneal Sarcoma: Diagnosis, Prognostic Factors and Treatment

**DOI:** 10.3390/cancers13164016

**Published:** 2021-08-10

**Authors:** Fabio Carbone, Antonio Pizzolorusso, Giuseppe Di Lorenzo, Massimiliano Di Marzo, Lucia Cannella, Maria Luisa Barretta, Paolo Delrio, Salvatore Tafuto

**Affiliations:** 1 Department of Advanced Biomedical Sciences, Università degli Studi di Napoli—“Federico II”, 80131 Naples, Italy; fa.carbone87@gmail.com; 2 Sarcomas and Rare Tumors Unit, Istituto Nazionale Tumori, IRCCS—Fondazione “G. Pascale”, 80131 Naples, Italy; a.pizzolorusso@istitutotumori.na.it (A.P.); giuseppedilorenzo10@gmail.com (G.D.L.); l.cannella@istitutotumori.na.it (L.C.); s.tafuto@istitutotumori.na.it (S.T.); 3 Abdominal Surgery Unit, Istituto Nazionale Tumori, IRCCS—Fondazione “G. Pascale”, 80131 Naples, Italy; p.delrio@istitutotumori.na.it; 4 Radiology Unit, Istituto Nazionale Tumori, IRCCS—Fondazione “G. Pascale”, 80131 Naples, Italy; m.barretta@istitutotumori.na.it

**Keywords:** retroperitoneal sarcoma, cancer, multidisciplinary, management, literature review

## Abstract

**Simple Summary:**

The management of retroperitoneal sarcomas can be challenging due to the variety of their presentation, histopathological types, and behaviours. This literature review provides a comprehensive and practical overview of the management of retroperitoneal sarcomas, focusing on diagnostic challenges, prognostic factors, multidisciplinary aspects of treatment and new research perspectives.

**Abstract:**

Retroperitoneal sarcomas (RPS) are rare cancers whose management can be challenging due to various presentation patterns, multiple organ involvement, and a high local and distant recurrence rate. Histopathology and prognostic factors analysis are essential to predict the behaviour of the disease and plan the best therapeutic strategy. To date, surgery is still the main therapeutic option that guarantees a chance of cure from the primary disease. While chemotherapy and radiotherapy seem to be good options for controlling metastatic and recurrent irresectable disease, their role in the treatment of primary RPS remains unclear. This literature review aims to provide a comprehensive overview of the multidisciplinary aspects of RPS management in high-volume centres, summarising the diagnostic path, the prognostic factors, and the most suitable therapeutic options.

## 1. Introduction

Retroperitoneal soft-tissue sarcomas (RPS) are rare tumours of mesenchymal origin. The correct incidence is difficult to establish: the crude incidence rate is 0.31 per 100,000 people per year [1]. About 53–56% of patients are female and the median age at diagnosis is 59–61 years old [2,3].

Only 16% of all sarcomas are located in the retroperitoneum, as they occur more commonly in the extremities (about 40%) [4]. Approximately one-third of the retroperitoneal masses are RPS, whereas other tumours could arise from retroperitoneal organs [5]. The variety of anatomical site of onset and histopathological types can make the diagnosis of the disease challenging. To date, the only possibility of cure and achievement of disease clearance is surgery, although the advancement of adjuvant therapies makes this pathology management framed in a multidisciplinary setting [6]. This literature review aims to provide an updated overview of the diagnosis and management of RPS.

## 2. Diagnosis

Presenting symptoms are often not specific and dependent on the anatomical site involved. The RPS usually grows as a mass, causing compression symptoms on other organs and a sense of abdominal discomfort, especially when it reaches a considerable volume. More frequently, RPS are incidental findings at the imaging tests performed for other reasons. Some of the most frequent symptoms are abdominal pain and discomfort, back pain, bowel obstruction, urinary and gynaecological symptoms. When the mass becomes bulky, it can be palpated externally [6,7].

### 2.1. Imaging and Guided Biopsies

A correct evaluation of the diagnostic images is paramount to stage the disease, establish the best therapeutic pathway and evaluate the surgical resectability. The contrast-enhanced computed tomography (CT) is the most valuable primary exam largely available that permits the diagnosis of retroperitoneal masses and the disease staging. The evaluation of the margins and the distortion of the other retroperitoneal organs confirm the retroperitoneal location [8]. About 21% of the lesions diagnosed as RPS at the CT scan turn out to be non-mesenchymal tumours at histopathology. Moreover, it has been shown that the CT alone is not able to provide the correct histopathological subtype, except for well-differentiated liposarcoma (WDLS) and angiomyolipoma [9,10]. The WDLS is constituted by well-differentiated hypodense fat, whereas the angiomyolipoma presents vascular structures in the fatty tissue. The finding of high-density areas in the contest of a fatty mass makes liposarcoma diagnosis even more likely. All high-density masses with no fatty component need a biopsy to differentiate soft-tissue sarcomas from other tumours (germ-cell tumours, lymphoma or desmoid) [11]. The CT scan of the chest and abdomen is important for staging the disease and detecting the presence of lung and liver metastases [7,12,13]. 

Magnetic resonance imaging (MRI) can assist in doubt on muscles, bones, foramina, and neurovascular structures involvement. It is essential to assess pelvic masses extent and evaluate the indication for radiotherapy and its treatment volume [14]. If the surgery involves the removal of a kidney, a functional examination of the contralateral could be considered.

The image-guided percutaneous core needle biopsy (CNB, 14–16 gauge) is essential for the histologic and molecular characterisation of the retroperitoneal mass, allowing the differential diagnosis between primary soft-tissue RPS, other malignant lesions, metastatic disease, and benign masses. It should always be performed unless images are pathognomonic of WDLS or the procedure is dangerous due to the proximity of the mass to vital anatomical structures. The safest retroperitoneal route is preferred, but the transperitoneal route can be considered when the latter is not feasible and a transperitoneal approach is considered safe [15]. The risk of needle tract seeding after percutaneous CNB is potentially possible but weak, amounting to 0.37–2%. It seems to be lower with the retroperitoneal route than the transperitoneal one. CNB is a safe procedure that does not impact the local recurrence and overall survival (OS) rates [16,17,18]. 

The CNB has recently shown to have 98% of specificity and 85% of positive predictive value in identifying high-grade RPS, leading to better identification of patients who may eventually benefit from preoperative neoadjuvant therapy [19]. The fine needle aspiration cytology (FNAC) and the endoscopic ultrasound-guided biopsy are rare options to be considered in specific circumstances [20,21]. 

On the one hand, liver and lung lesions with metastasis features on imaging in the context of a biopsy-confirmed primary RPS do not require a histological diagnosis. On the other hand, lesions with atypical radiological characteristics for sarcoma metastases in atypical sites and in the context of multiple primary tumours require histological sampling [22]. In synchronous metastatic disease, a biopsy of the metastasis could be considered a priority over that of the primary.

The fluorodeoxyglucose positron emission tomography (FDG PET-CT) is not used routinely, as it is not able to distinguish benign and malignant retroperitoneal tumours. However, the standardised uptake value (SUV) is higher in high-grade/undifferentiated tumours than low-grade and benign ones. Indeed, the SUV correlates with the mitotic count, ki-67 index, histological grade and recurrent RPS. The maximum SUV location can guide the needle biopsy towards the most avid component of the mass [23,24]. 

The staging system currently used for RPS is the American Joint Committee on Cancer (AJCC) stage classification system, 8th edition, shown in Table 1 [25]. 

### 2.2. Surgical Biopsy

Surgical biopsy by laparoscopy or laparotomy should be avoided as it exposes the patient to an operation with the possible spread of tumour cells, risk of damaging neurovascular structures and compromising further surgery. Moreover, the sample taken may not be representative of the mass on histological examination as it would be harvested not under imaging guidance. The use of surgical biopsy should therefore be limited to those cases where a CT- or ultrasound-guided CNB is not feasible [11]. 

### 2.3. Histopathology

Over 75 histologic types of soft-tissue sarcoma can occur in the retroperitoneum, each with different behaviour [26,27]. The correct histologic subtype is usually individualised with immunohistochemical staining and can be further confirmed by molecular biology techniques, such as fluorescence in situ hybridization or reverse transcriptase-polymerase chain reaction that are used to detect sarcoma-specific gene mutations and mRNA differentiation. Therefore, a dedicated pathology unit would be required in referral centres for soft-tissue sarcoma management.

Among RPS, the most frequent histologic types are liposarcoma (about 56.8%), leiomyosarcoma (LMS, 24.7%) and undifferentiated sarcoma (8.6%). Liposarcomas are subdivided into well-differentiated, dedifferentiated (DDLS), myxoid (ML), pleomorphic, mixed and not otherwise specified [3]. Table 2 shows the principal histologic types and grades of RPS and their frequencies in some series [3,28,29,30,31,32,33,34]. 

The most used grading systems for soft-tissue sarcomas are the French Federation of Cancer Centers Sarcoma Group (FNCLCC) [35] and the National Cancer Institute (NCI) [36], both are three-grade systems.

## 3. Prognostic Factors

The 5-year OS for patients with treated RPS is 64–72%, and the cumulative incidence of LR and DM is 24–39% and 21–24%, respectively [29,31,37,38]. Long-term survival outcomes increased over the past fifteen years due to a better selection of patients for surgery, perioperative care, and surgical resection quality [39]. Many studies aimed to identify prognostic factors associated with the OS and the disease-free survival (DFS) of patients with RPS.

The identified prognostic factors associated with the OS are age, gender, tumour size, number of organs resected, invasion of adjacent structures, radicality of the surgical resection, multifocality, histopathological subtype and grade [29,30,31,37,38]. Gender, size of the tumour, histologic grade, completeness of surgical resection margins, adjacent organ involvement, specialisation of the surgeon, piecemeal resection and perioperative radiotherapy are associated with LR, whereas histology grade, subtype and adjacent organ involvement are associated with DM. Specialisation of the surgeon and piecemeal resection are prognostic factors associated with abdominal sarcomatosis [37,40]. 

Tan et al. demonstrated that the histologic type is a significant independent prognostic factor of disease-specific death (DSD), local recurrence (LR) and distant metastases (DM), able to predict patterns of recurrence in patients who have undergone resection surgery. WDLS and ML are related to better specific survival (DSD risk of 25% at 10 years) compared to solitary fibrous tumours (SFT, DSD risk of 34%), DDLS, round cell and pleomorphic liposarcomas (DSD risk 53%), malignant peripheral nerve sheath tumours (MPNST, DSD risk 56%) and high-grade LMS (DSD risk 60%). WDLS and ML have a 5-year LR rate of 39% and a 15-years LR rate of 60%; MPNST and SFT have a 3-year LR rate of 35% and 8%, respectively, with no increase in the following years. High-grade LMS, SFT and DDLS are associated with the highest DM incidence (10-year DM rates of 58%, 41% and 28%, respectively). MPNST and WDLS are associated with a low DM risk (10-year DM rate of 15% and 8%, respectively) [29]. 

Another predictor of worse OS is the RPS histopathologic organ invasion, which seems not to be associated with an increased incidence of LR and DM. [41] Histology-related patterns of recurrence are shown in Table 3. Based on the analysis above, the importance of treating the RPSs in highly dedicated and specialised centres is evident. Furthermore, the postoperative follow-up surveillance strategies should consider the tumour biology to predict eventual recurrences and not lose any potential opportunity for successful salvage therapy [27]. 

## 4. Treatment

A dedicated multidisciplinary team should carry out the management of RPS in high-volume centres and the treatment should be discussed on a case-by-case assessment [12]. 

### 4.1. Radiotherapy

The efficacy of neoadjuvant radiotherapy (RT) for RPS has always been controversial and is still under investigation. Some of the neoadjuvant RT treatment advantages under investigation are tumour debulking, increased chances of having a disease-free resection margin after radical surgery, and eventual thickening of the tumour capsule, leading to more radical surgery [42]. Concerns about preoperative RT would eventually be the damage caused to the bowel and nearby structures, as well as an increased rate of postoperative complications. The first studies on preoperative RT showed a high rate of patients that failed to undergo curative surgery due to disease progression or treatment complications [43]. Radiotherapy technologies, such as intensity-modulated RT, stereotactic ablative RT, brachytherapy, and intra-operative RT (IORT) have contributed to reducing the radiation dose on normal tissues surrounding the tumour. Although the IORT has recently shown promising results in local control of the disease, its role still appears limited due to concerns about its toxicity [44]. Brachytherapy is also associated with high toxicity and its value is unproven [45]. 

Preoperative radiotherapy with selective augmentation on the margin at the highest risk of local recurrence appeared to be a safe tool. It was advantageous when the tumour was considered unresectable or marginally resectable due to the risk of positive margins after surgery [46].

However, the survival benefit of neoadjuvant RT is still under investigation. The first studies failed to demonstrate a clear benefit of preoperative RT on the OS [43,47]. Instead, in a propensity score-matched analysis of 9068 patients from the National Cancer Database, both the preoperative RT (HR 0.70, 95% CI 0.59–0.82, *p* < 0.001) and the postoperative RT (HR 0.78, 95% CI 0.71–0.85, *p* < 0.0001) were associated with a better OS compared with surgery alone [48]. A recent population-based study highlighted the correlation between the use of neoadjuvant RT followed by surgery and negative microscopic resection margins, with better LR-free survival (HR 0.43, 95% CI 0.24–0.79, *p* = 0.01 on the multivariate analysis) and better OS (HR 0.42, 95% CI 0.19–0.90, *p* = 0.03 on the multivariate analysis) in comparison with surgery alone. [49] To date, the only multicentre, randomised, phase 3 trial comparing radiotherapy followed by surgery versus surgery alone is the STRASS-1 trial. It showed a higher rate of severe complications in the RT plus surgery group than in the surgery alone one (24% versus 10% respectively). The mortality rate for treatment-related adverse events was 1% in the RT plus surgery group and 0% in the surgery alone one. No difference was noted between the groups regarding postoperative reoperation rate (11%) and postoperative mortality rate (2%). Furthermore, no difference was noted in terms of abdominal recurrence-free survival (HR 1.01, 95% CI 0.71–1.44, *p* = 0.95) and OS (HR 1.16, 95% CI 0.65–2.05, *p* = 0.62) between surgery alone versus RT plus surgery groups. The trial concluded that the preoperative RT should not be considered as the standard of care treatment for RPS [50]. 

Adjuvant postoperative RT has been shown to have a small benefit on local disease control, other than by delaying LR [51,52]. Therefore, postoperative RT has been progressively abandoned due to the high morbidity rate, the poor benefit on cancer control and the growing interest in preoperative RT protocols.

### 4.2. Chemotherapy

An early neoadjuvant systemic therapy aims to assess the tumour response, modulate the treatment, and reduce the possibility of micro-metastases formation in sarcomas with prevalent blood diffusion, such as high-grade dedifferentiated liposarcoma, leiomyosarcoma and undifferentiated pleomorphic sarcoma, which seem to be chemosensitive [53]. Since soft-tissue sarcomas’ response to systemic chemotherapy is relatively poor (around 16–27% of metastatic patients are responsive to doxorubicin therapy), the concern is to delay the radical surgery unnecessarily [54]. Indeed, since the prognosis is generally given by the probability of local recurrence, rather than by the rarer probability of distant metastases, a delay in surgery could negatively impact the prognosis. However, the tumour downsizing and the pathological response to chemotherapy could increase the likelihood of the surgery being radical [42].

Doxorubicin, alone or associated with ifosfamide, is the most used agent. The combined therapy does not improve the OS, but improves the objective response rate in patients with locally advanced, unresectable, or metastatic high grade soft-tissue sarcoma (26% of patients in combined therapy versus 14% in doxorubicin alone). However, higher toxicity, especially myelosuppression, is shown in the combination therapy [55]. There are many ongoing prospective trials comparing chemotherapy, immune inhibitors, and molecular target agents [26]. A prospective multicentre randomised trial (STRASS-2, NCT04031677) started in 2019 intending to compare the outcomes of surgery with or without neoadjuvant chemotherapy in high-risk RPS (high-grade liposarcoma and leiomyosarcoma) [56]. To date, there are no randomised controlled trials that compare neoadjuvant chemotherapy/chemoradiotherapy with surgery alone.

The multicentre randomized controlled trial EORTC 62931 showed that chemotherapy after soft-tissue sarcoma resection does not improve OS and DFS [57].

Furthermore, some studies showed chemotherapy could play a role in the radio-sensitisation of RPS, which could therefore benefit more from RT. The combination of three cycles of ifosfamide (14 gr/m^2^) and RT (up to 50.4 Gy), followed by surgery after 4–6 weeks, is feasible and safe, but it is not proven yet to be better than other strategies due to lack of data [58]. 

Based on the results above, chemotherapy has no role in the management of RPS outside from clinical trials.

### 4.3. Surgery

Surgery remains the only possible treatment that offers a chance of radicalisation and cure from the disease. The correct surgery consists of en-bloc resection of the tumour with the removal of all the structures involved. Extended surgery with a free-tumour margin offers the best results in terms of LR rates compared with simple excisions. The best chance for a curative resection is at the time of the primary presentation of the RPS. Resection of specific organs should be performed to ensure a disease-free resection margin, and the choice of which structures to resect should be made considering the long-term dysfunction caused, the chances of complete resection and the patient’s life expectancy. Bilateral renal involvement, superior mesenteric artery, celiac tripod, and portal vein infiltration, as well as spinal cord involvement, are considered contraindications to surgery [6,11,12,59]. 

Careful preoperative evaluation using MRI and CT scan images, as well as three-dimensional reconstructions, is critical to plan the margins of the resection and anticipate the structures and organs involved in the excision. The histology of the RPS must be considered in the preoperative multidisciplinary setting, as histopathological subtypes have different organ involvement and local/distant recurrence patterns after resection surgery [29,31,37]. Furthermore, histopathologic organ invasion is considered a predicting factor of OS [41]. Therefore, planning and extension of surgery should also be guided by the histological type. Liposarcomas, especially if well-differentiated, do not generally have clear margins and the fatty tissue is not distinguishable from the retroperitoneal fat. In these cases, more extensive resections may be indicated. Instead, LMS and SFT have more defined margins, and therefore if adjacent organs are not infiltrated, they could be spared [6]. The involvement of major vessels is not a contraindication to radical surgery, although major vascular resection is associated with higher morbidity [60,61]. 

Since the surgery of retroperitoneal sarcomas can include multiorgan resections, major vascular resections with or without reconstruction and removal of muscles and bones with the need to save vascular bundles, it should be performed in dedicated high-volume centres by different surgical teams with specific expertise in different anatomical regions and organs.

Cytoreductive surgery with hyperthermic intraperitoneal intraoperative chemotherapy (HIPEC) for abdominal multifocal sarcomatosis is associated with a high toxicity rate without conferring any survival advantage [62,63,64]. A complete cytoreduction associated with HIPEC could effectively treat patients with uterine sarcomas and desmoplastic small round cell tumour [65,66,67]. However, the role of HIPEC is still under investigation.

## 5. Metastatic Disease

In patients with RPS metastatic disease, a tailored treatment should be designed in a multidisciplinary setting. Surgical and systemic treatment options should consider the histopathological subtype and its behaviour, as well as the patient’s symptoms and status. In general, the complete radicalisation of the disease with resection surgery is the treatment that leads to the best long-term survival outcome. In patients with metastases, the primary surgery can be performed in selected cases to reduce the local disease burden or practice a complete local radicalisation, reduce symptoms, and facilitate any resection surgery on possible recurrences.

Surgery on liver or lung metastases can be considered with the sense of completely radicalising the disease in selected patients with good performance status and a high life expectancy. Patient selection based on favourable tumour biology should consider the low-volume disease, DFS time greater than 12 months, and response or prolonged stability to systemic chemotherapy [22]. Local therapies, such as radiofrequency or microwave ablations, can be important in the resection strategy and control of the disease, variously combined with surgery [68,69]. 

Metachronous lung metastases (DFS ≥ 1 year) can be resected if radicalisation of the disease can be achieved [70]. Synchronous lung metastases should be treated with chemotherapy, reserving surgery for resectable residual lung lesions [71]. Extrapulmonary disease is not a contraindication to curative multiorgan resection, as long the radicalisation can be achieved, and the patient’s status is adequate. Extrapulmonary metastases can be treated with chemotherapy first, and surgery should be offered for responding metastases in selected patients [53]. 

In large-volume liver metastatic disease, arterial embolisation or chemoembolisation can be considered [72,73]. 

Intra-abdominal multifocal metastases can be treated with surgical resections, which may confer symptoms control, but incomplete resections do not have any benefit on survival. The recurrent metastatic disease should be approached with surgery only if the biology of the tumour is favourable: low-grade histology, low-volume disease (in number and size) and high DFS time [74,75]. RT could be an option for palliation of pain or symptoms of spinal compression.

Chemotherapy is usually the first approach in synchronous metastatic disease or non-resectable disease, especially if poor prognostic factors are present (high grade and high number of lesions). Administration of chemotherapy before surgery helps assess the response and modulate the treatment: regression or stable disease over 6 months may be a good factor for considering surgery [22,53,76]. In unresectable metastatic disease, systemic therapy should aim at retarding the growth of the mass, prolonging life expectancy as much as possible and ensuring a decent quality of life.

Anthracycline-based chemotherapy (doxorubicin or epirubicin) is the first-line treatment and the association with ifosfamide or dacarbazine can be considered [77,78,79,80]. A combined therapy with dacarbazine is preferred for LMS and SFT [81,82]. Recently the phase 3 ANNOUNCE trial showed there was no difference in OS with the addition of olaratumab to doxorubicin [83]. More agents can be considered as a second-line treatment, or in case anthracyclines are contraindicated (Table 4) [22].

## 6. Recurrent Disease

After primary resection, local recurrence of RPS is common and is generally associated with a worse prognosis than primary RPS. As mentioned before, the different histological subtypes are associated with different recurrence patterns over time (Table 2) [37]. At the time of the recurrence staging, it would be advisable to perform a percutaneous core biopsy in order to confirm the actual relapse and possibly target the therapy [13].

A multidisciplinary evaluation of each case should be carried out to evaluate the long-term prognosis and the chance of disease-free survival, to design an appropriate therapy.

In unifocal locoregional recurrence, a curative resection can be considered when complete excision can be guaranteed [100]. In case of recurrent multifocal abdominal disease, radical excision of the disease is unlikely, and surgery should be performed with palliative intent [101,102,103]. Therefore, a correct selection of patients for surgery is essential and should be based on the number of local recurrences, histopathologic subtype, grade, and rate of tumour growth: unifocal recurrences, histology of WDLS, low grade and growth rate of less than 1 cm per month are associated with better survival [104]. Instead, in the case of synchronous abdominal and distant recurrences, the patient should be considered for systemic therapy rather than for surgery [40]. However, neoadjuvant therapy before surgery could be considered for all patients with recurrent disease to downsize the tumour and assess its responsiveness to medical therapies [13]. The efficacy of postoperative adjuvant therapies in fully resected recurrent disease has not been proven [45,52,57]. Patients with the unresectable recurrent disease could benefit from systemic therapy, RT for symptoms control and, in selected cases, palliative surgery [13,105].

## 7. Follow-Up

The follow-up schedule should be set up considering the likelihood that the disease will recur.

The postoperative follow-up for patients at high/intermediate risk of recurrence should be performed with CT scan of the lung and abdomen and MRI scan of the abdomen every 3–4 months for the first 2–3 years, then every 6 months for the next 3 years and once a year afterwards [53]. Patients at low risk of recurrence can be followed up every 4–6 months for the first 3–5 years, then once a year. A 5-year follow-up period seems insufficient, as recent evidence has shown that approximately 9% of local recurrences and 6% of distant recurrences occur later, after 5 years from the operation [27,31,106]. Therefore, the follow-up period should be at least 10 years or even indefinite [12,15].

## 8. Conclusions

Retroperitoneal sarcomas are rare soft tissue tumours that can be difficult to manage due to the variety of behaviours and locations they may have. To date, radical surgery, possibly extended to multiple organs, remains the only possibility of curative treatment of the disease. Radiotherapy and chemotherapy appear to have an adjuvant role, especially in controlling recurrent and metastatic disease, although further studies are needed. The correct selection of patients to address the different therapeutic pathways remains a crucial point, which is why a dedicated multidisciplinary team must evaluate the treatment of retroperitoneal sarcomas in high volume centres.

## Figures and Tables

**Table 1:** The American Joint Committee on Cancer (AJCC) stage classification system, 8th edition for retroperitoneal sarcomas.

Retroperitoneal Soft-Tissue Sarcoma AJCC—TNM 8th Edition Staging System	
Tx	Primary tumour cannot be assessed
T0	No evidence of primary tumour
T1	Tumour ≤ 5 cm in greatest dimension
T2	Tumour > 5 cm and ≤ 10 cm
T3	Tumour > 10 cm and ≤15 cm
T4	Tumour > 15 cm
N0	No regional lymph node metastasis or unknown lymph node status
N1	Regional lymph node metastasis
M0	No distant metastasis
M1	Distant metastasis

**Table 2:** Numbers and ratios of the most common retroperitoneal sarcoma histologic types and grades in some recent series.

Histological Types and Grades	Total		Huggett 2019 [3]		Raut 2019 [28]		Tan 2016 [29]		Garcia-Ortega 2016 [30]		Gronchi 2016 [31]		Gronchi 2013 [32]		Nathan 2009 [33]		van Dalen 2004 [34]	
	*n*	*%*	*n*	*%*	*n*	*%*	*n*	*%*	*n*	*%*	*n*	*%*	*n*	*%*	*n*	*%*	*n*	*%*
**Total**	11354	100	6857	100	602	100	674	100	95	100	1007	100	523	100	1365	100	231	100
**LS**	6446	56.8	3857	56.3	435	72.3	399	59.2	58	61.1	633	62.8	276	52.7	682	49.9	106	45.9
WDLS	2077	18.3	1311	19.1	169	28.1	186 *	27.6 *	27 *	28.4 *	263	26.1	121	23.1				
DDLS	2494	22.0	1459	21.3	266	44.2	213 **	31.6 **	31 **	32.6 **	370	36.7	155	29.6				
**LMS**	2802	24.7	1868	27.2	73	12.1	150	22.3	14	14.7	194	19.3	92	17.6	358	26.2	53	22.9
**US**	974	8.6	776	11.3							22	2.2	70	13.4				
**SFT**	206	1.8	74	1.1	14	2.3	33	4.9			59	5.8	26	5.0				
**MPNST**	185	1.6	86	1.3	7	1.2	23	3.4			33	3.3	16	3.1	15	1.1	5	2.2
**FS**	108	0.9	71	1.0											24	1.8	13	5.6
**OHT**	633	5.6	125	1.8	73	12.1	69	10.2	23	24.2	66	6.6	43	8.2	286	21.0	54	23.4
**Low-grade**	3515	31.0	2110	30.8	195	32.4	242	35.9	29	30.5	329	32.7	147	28.1	585	42.9	103	44.6
**Intermediate grade**	2349	20.7	1203	17.6	170	28.2			2	2.1	370	36.7	122	23.3	213	15.6	44	19.0
**High-grade**	4729	41.6	3103	45.2	237	39.4	431	64.0	64	67.4	267	26.5	254	48.6	292	21.4	81	35.1
**Grade not specified**	761	6.7	441	6.4			1	0.1			41	4.1			275	20.1	3	1.3

LS: liposarcoma; WDLS: well-differentiated liposarcoma; DDLS: dedifferentiated liposarcoma; LMS: leiomyosarcoma; US: undifferentiated sarcoma; SFT: solitary fibrous tumour; MPNST: malignant peripheral nerve sheath tumour; OHT: other histology types; FS: fibrosarcoma. Histologic grade according to the Federation Nationale des Centres de Lutte Contre le Cancer (FNCLCC). * Myxoid liposarcoma is included. ** Round cell and pleomorphic liposarcoma are included.

**Table 3:** Risk of local recurrence and distant metastases of the most common retroperitoneal sarcoma histologic types according to two recent series [29,37]. Early recurrence: ≤5 years from the operation; late recurrence: 5–15 years from the operation [29].

Histological Types	Local Recurrence		Distant Metastases	
	Early	Late	Early	Late
WDLS	18–39%	60%	0%	8%
DDLS	33–58%	62%	9–44%	28%
LMS	6–16%	24%	55%	58%
SFT	4–8%		17%	41%
MPNST	20–35%		12%	15%

WDLS: well-differentiated liposarcoma; DDLS: dedifferentiated liposarcoma; LMS: leiomyosarcoma; SFT: solitary fibrous tumour; MPNST: malignant peripheral nerve sheath tumour.

**Table 4:** Chemotherapy regimens for metastatic or irresectable retroperitoneal sarcomas.

Therapy Lines	Chemotherapy	Histologic Subtype-Specific Indication
First-line	Doxorubicin/Epirubicin [55,79]	-
	Doxorubicin + Ifosfamide [55,79]	-
	Dacarbazine ± Doxorubicin [84,85]	LMS, SFT
Second-line	Ifosfamide [86,87]	DDLS, MPNST
	Trabectedin [88,89]	LS, LMS
	Eribulin [90,91]	LS
	Gemcitabine ± Docetaxel (or Dacarbazine) [92,93,94,95,96]	LMS, UPS
	Pazopanib [97,98]	non-LS
	Sunitinib, Temozolomide [85]	SFT
	Sirolimus [99]	PEComa

LS: liposarcoma; DDLS: dedifferentiated liposarcoma; LMS: leiomyosarcoma; SFT: solitary fibrous tumour; MPNST: malignant peripheral nerve sheath tumour; UPS: undifferentiated pleomorphic sarcoma.

## References

[1] Gatta G., Capocaccia R., Botta L., Mallone S., De Angelis R., Ardanaz E., Comber H., Dimitrova N., Leinonen M.K., Siesling S. (2017). Burden and centralised treatment in Europe of rare tumours: Results of RARECAREnet-a population-based study. Lancet Oncol..

[2] Wiseman J.T., Ethun C.G., Cloyd J.M., Shelby R., Suarez-Kelly L., Tran T., Poultsides G., Mogal H., Clarke C., Tseng J. (2020). Analysis of textbook outcomes among patients undergoing resection of retroperitoneal sarcoma: A multi-institutional analysis of the US Sarcoma Collaborative. J. Surg. Oncol..

[3] Huggett B.D., Cates J.M.M. (2019). The Vanderbilt staging system for retroperitoneal sarcoma: A validation study of 6857 patients from the National Cancer Database. Mod. Pathol..

[4] Brennan M.F., Antonescu C.R., Moraco N., Singer S. (2014). Lessons learned from the study of 10,000 patients with soft tissue sarcoma. Ann. Surg..

[5] Van Roggen J.F., Hogendoorn P.C. (2000). Soft tissue tumours of the retroperitoneum. Sarcoma.

[6] Dumitra S., Gronchi A. (2018). The Diagnosis and Management of Retroperitoneal Sarcoma. Oncology (Williston Park).

[7] Messiou C., Moskovic E., Vanel D., Morosi C., Benchimol R., Strauss D., Miah A., Douis H., van Houdt W., Bonvalot S. (2017). Primary retroperitoneal soft tissue sarcoma: Imaging appearances, pitfalls and diagnostic algorithm. Eur. J. Surg. Oncol..

[8] Tirkes T., Sandrasegaran K., Patel A.A., Hollar M.A., Tejada J.G., Tann M., Akisik F.M., Lappas J.C. (2012). Peritoneal and retroperitoneal anatomy and its relevance for cross-sectional imaging. Radiographics.

[9] Morosi C., Stacchiotti S., Marchianò A., Bianchi A., Radaelli S., Sanfilippo R., Colombo C., Richardson C., Collini P., Barisella M. (2014). Correlation between radiological assessment and histopathological diagnosis in retroperitoneal tumors: Analysis of 291 consecutive patients at a tertiary reference sarcoma center. Eur. J. Surg. Oncol..

[10] Woo S., Kim S.Y., Cho J.Y., Kim S.H., Lee M.S. (2016). Exophytic renal angiomyolipoma and perirenal liposarcoma: Revisiting the role of CT for differential diagnosis. Acta Radiol..

[11] Bonvalot S., Raut C.P., Pollock R.E., Rutkowski P., Strauss D.C., Hayes A.J., Van Coevorden F., Fiore M., Stoeckle E., Hohenberger P. (2012). Technical considerations in surgery for retroperitoneal sarcomas: Position paper from E-Surge, a master class in sarcoma surgery, and EORTC-STBSG. Ann. Surg. Oncol..

[12] Trans-Atlantic RPS Working Group (2015). Management of primary retroperitoneal sarcoma (RPS) in the adult: A consensus approach from the Trans-Atlantic RPS Working Group. Ann. Surg. Oncol..

[13] Trans-Atlantic RPS Working Group (2016). Management of Recurrent Retroperitoneal Sarcoma (RPS) in the Adult: A Consensus Approach from the Trans-Atlantic RPS Working Group. Ann. Surg. Oncol..

[14] White L.M., Wunder J.S., Bell R.S., O’Sullivan B., Catton C., Ferguson P., Blackstein M., Kandel R.A. (2005). Histologic assessment of peritumoral edema in soft tissue sarcoma. Int. J. Radiat. Oncol. Biol. Phys..

[15] Messiou C., Morosi C. (2018). Imaging in retroperitoneal soft tissue sarcoma. J. Surg. Oncol..

[16] Berger-Richardson D., Swallow C.J. (2017). Needle tract seeding after percutaneous biopsy of sarcoma: Risk/benefit considerations. Cancer.

[17] Van Houdt W.J., Schrijver A.M., Cohen-Hallaleh R.B., Memos N., Fotiadis N., Smith M.J., Hayes A.J., Van Coevorden F., Strauss D.C. (2017). Needle tract seeding following core biopsies in retroperitoneal sarcoma. Eur. J. Surg. Oncol..

[18] Wilkinson M.J., Martin J.L., Khan A.A., Hayes A.J., Thomas J.M., Strauss D.C. (2015). Percutaneous core needle biopsy in retroperitoneal sarcomas does not influence local recurrence or overall survival. Ann. Surg. Oncol..

[19] Almond L.M., Tirotta F., Tattersall H., Hodson J., Cascella T., Barisella M., Marchianò A., Greco G., Desai A., Ford S.J. (2019). Diagnostic accuracy of percutaneous biopsy in retroperitoneal sarcoma. Br. J. Surg..

[20] Gupta P., Rajwanshi A., Nijhawan R., Srinivasan R., Gupta N., Saikia U.N., Dey P. (2017). Fine needle aspiration in retroperitoneal lesions. APMIS.

[21] Sofi A.A., Thekdi A.D., Nawras A. (2011). EUS-FNA for the Diagnosis of Retroperitoneal Primitive Neuroectodermal Tumor. Diagn. Ther. Endosc..

[22] Trans-Atlantic Retroperitoneal Sarcoma Working Group (TARPSWG) (2018). Management of metastatic retroperitoneal sarcoma: A consensus approach from the Trans-Atlantic Retroperitoneal Sarcoma Working Group (TARPSWG). Ann. Oncol..

[23] Liu D.N., Li Z.W., Wang H.Y., Zhao M., Zhao W., Hao C.Y. (2018). Use of 18F-FDG-PET/CT for Retroperitoneal/Intra-Abdominal Soft Tissue Sarcomas. Contrast Media Mol. Imaging.

[24] Ioannidis J.P., Lau J. (2003). 18F-FDG PET for the diagnosis and grading of soft-tissue sarcoma: A meta-analysis. J. Nucl. Med..

[25] Gospodarowicz M.K., Brierley J.D., Wittekind C. (2016). TNM Classification of Malignant Tumours.

[26] Sassa N. (2020). Retroperitoneal tumors: Review of diagnosis and management. Int. J. Urol..

[27] Zaidi M.Y., Canter R., Cardona K. (2018). Post-operative surveillance in retroperitoneal soft tissue sarcoma: The importance of tumor histology in guiding strategy. J. Surg. Oncol..

[28] Raut C.P., Callegaro D., Miceli R., Barretta F., Rutkowski P., Blay J.Y., Lahat G., Strauss D.C., Gonzalez R., Ahuja N. (2019). Predicting Survival in Patients Undergoing Resection for Locally Recurrent Retroperitoneal Sarcoma: A Study and Novel Nomogram from TARPSWG. Clin. Cancer Res..

[29] Tan M.C., Brennan M.F., Kuk D., Agaram N.P., Antonescu C.R., Qin L.X., Moraco N., Crago A.M., Singer S. (2016). Histology-based Classification Predicts Pattern of Recurrence and Improves Risk Stratification in Primary Retroperitoneal Sarcoma. Ann. Surg..

[30] Garcia-Ortega D.Y., Villa-Zepeda O., Martinez-Said H., Cuellar-Hübbe M., Luna-Ortiz K. (2016). Oncology outcomes in Retroperitoneal sarcomas: Prognostic factors in a Retrospective Cohort study. Int. J. Surg..

[31] Gronchi A., Strauss D.C., Miceli R., Bonvalot S., Swallow C.J., Hohenberger P., Van Coevorden F., Rutkowski P., Callegaro D., Hayes A.J. (2016). Variability in Patterns of Recurrence After Resection of Primary Retroperitoneal Sarcoma (RPS): A Report on 1007 Patients from the Multi-institutional Collaborative RPS Working Group. Ann. Surg..

[32] Gronchi A., Miceli R., Shurell E., Eilber F.C., Eilber F.R., Anaya D.A., Kattan M.W., Honoré C., Lev D.C., Colombo C. (2013). Outcome prediction in primary resected retroperitoneal soft tissue sarcoma: Histology-specific overall survival and disease-free survival nomograms built on major sarcoma center data sets. J. Clin. Oncol..

[33] Nathan H., Raut C.P., Thornton K., Herman J.M., Ahuja N., Schulick R.D., Choti M.A., Pawlik T.M. (2009). Predictors of survival after resection of retroperitoneal sarcoma: A population-based analysis and critical appraisal of the AJCC staging system. Ann. Surg..

[34] Van Dalen T., Hennipman A., Van Coevorden F., Hoekstra H.J., Van Geel B.N., Slootweg P., Lutter C.F.A., Brennan M., Singer S. (2004). Evaluation of a Clinically Applicable Post-Surgical Classification System for Primary Retroperitoneal Soft-Tissue Sarcoma. Ann. Surg. Oncol..

[35] Trojani M., Contesso G., Coindre J.M., Rouesse J., Bui N.B., de Mascarel A., Goussot J.F., David M., Bonichon F., Lagarde C. (1984). Soft-tissue sarcomas of adults; study of pathological prognostic variables and definition of a histopathological grading system. Int. J. Cancer.

[36] Costa J., Wesley R.A., Glatstein E., Rosenberg S.A. (1984). The grading of soft tissue sarcomas. Results of a clinicohistopathologic correlation in a series of 163 cases. Cancer.

[37] Gronchi A., Miceli R., Allard M.A., Callegaro D., Le Péchoux C., Fiore M., Honoré C., Sanfilippo R., Coppola S., Stacchiotti S. (2015). Personalizing the approach to retroperitoneal soft tissue sarcoma: Histology-specific patterns of failure and postrelapse outcome after primary extended resection. Ann. Surg. Oncol..

[38] Chou Y.S., Liu C.Y., Chang Y.H., King K.L., Chen P.C., Pan C.C., Shen S.H., Liu Y.M., Lin A.T., Chen K.K. (2016). Prognostic factors of primary resected retroperitoneal soft tissue sarcoma: Analysis from a single asian tertiary center and external validation of gronchi’s nomogram. J. Surg. Oncol..

[39] Callegaro D., Raut C.P., Ng D., Strauss D.C., Honoré C., Stoeckle E., Bonvalot S., Haas R.L., Vassos N., Conti L. (2020). Has the Outcome for Patients Who Undergo Resection of Primary Retroperitoneal Sarcoma Changed Over Time? A Study of Time Trends During the Past 15 years. Ann. Surg. Oncol..

[40] Toulmonde M., Bonvalot S., Ray-Coquard I., Stoeckle E., Riou O., Isambert N., Bompas E., Penel N., Delcambre-Lair C., Saada E. (2014). Retroperitoneal sarcomas: Patterns of care in advanced stages, prognostic factors and focus on main histological subtypes: A multicenter analysis of the French Sarcoma Group. Ann. Oncol..

[41] Fairweather M., Wang J., Jo V.Y., Baldini E.H., Bertagnolli M.M., Raut C.P. (2017). Incidence and Adverse Prognostic Implications of Histopathologic Organ Invasion in Primary Retroperitoneal Sarcoma. J. Am. Coll Surg..

[42] Almond L.M., Gronchi A., Strauss D., Jafri M., Ford S., Desai A. (2018). Neoadjuvant and adjuvant strategies in retroperitoneal sarcoma. Eur. J. Surg. Oncol..

[43] Jones J.J., Catton C.N., O’Sullivan B., Couture J., Heisler R.L., Kandel R.A., Swallow C.J. (2002). Initial results of a trial of preoperative external-beam radiation therapy and postoperative brachytherapy for retroperitoneal sarcoma. Ann. Surg. Oncol..

[44] Stucky C.C., Wasif N., Ashman J.B., Pockaj B.A., Gunderson L.L., Gray R.J. (2014). Excellent local control with preoperative radiation therapy, surgical resection, and intra-operative electron radiation therapy for retroperitoneal sarcoma. J. Surg. Oncol..

[45] Smith M.J., Ridgway P.F., Catton C.N., Cannell A.J., O’Sullivan B., Mikula L.A., Jones J.J., Swallow C.J. (2014). Combined management of retroperitoneal sarcoma with dose intensification radiotherapy and resection: Long-term results of a prospective trial. Radiother Oncol..

[46] Tzeng C.W., Fiveash J.B., Popple R.A., Arnoletti J.P., Russo S.M., Urist M.M., Bland K.I., Heslin M.J. (2006). Preoperative radiation therapy with selective dose escalation to the margin at risk for retroperitoneal sarcoma. Cancer.

[47] Nussbaum D.P., Speicher P.J., Gulack B.C., Ganapathi A.M., Englum B.R., Kirsch D.G., Tyler D.S., Blazer D.G. (2015). Long-term Oncologic Outcomes After Neoadjuvant Radiation Therapy for Retroperitoneal Sarcomas. Ann. Surg..

[48] Nussbaum D.P., Rushing C.N., Lane W.O., Cardona D.M., Kirsch D.G., Peterson B.L., Blazer D.G. (2016). Preoperative or postoperative radiotherapy versus surgery alone for retroperitoneal sarcoma: A case-control, propensity score-matched analysis of a nationwide clinical oncology database. Lancet Oncol..

[49] Turner B.T., Hampton L., Schiller D., Mack L.A., Robertson-More C., Li H., Quan M.L., Bouchard-Fortier A. (2019). Neoadjuvant radiotherapy followed by surgery compared with surgery alone in the treatment of retroperitoneal sarcoma: A population-based comparison. Curr. Oncol..

[50] Bonvalot S., Gronchi A., Le Péchoux C., Swallow C.J., Strauss D., Meeus P., van Coevorden F., Stoldt S., Stoeckle E., Rutkowski P. (2020). Preoperative radiotherapy plus surgery versus surgery alone for patients with primary retroperitoneal sarcoma (EORTC-62092: STRASS): A multicentre, open-label, randomised, phase 3 trial. Lancet Oncol..

[51] Catton C.N., O’Sullivan B., Kotwall C., Cummings B., Hao Y., Fornasier V. (1994). Outcome and prognosis in retroperitoneal soft tissue sarcoma. Int. J. Radiat Oncol. Biol. Phys..

[52] Tseng W.H., Martinez S.R., Do L., Tamurian R.M., Borys D., Canter R.J. (2011). Lack of survival benefit following adjuvant radiation in patients with retroperitoneal sarcoma: A SEER analysis. J. Surg. Res..

[53] Casali P.G., Abecassis N., Aro H.T., Bauer S., Biagini R., Bielack S., Bonvalot S., Boukovinas I., Bovee J.V.M.G., Brodowicz T. (2018). Soft tissue and visceral sarcomas: ESMO-EURACAN Clinical Practice Guidelines for diagnosis, treatment and follow-up. Ann. Oncol..

[54] Sleijfer S., Seynaeve C., Verweij J. (2005). Using single-agent therapy in adult patients with advanced soft tissue sarcoma can still be considered standard care. Oncologist.

[55] Judson I., Verweij J., Gelderblom H., Hartmann J.T., Schöffski P., Blay J.Y., Kerst J.M., Sufliarsky J., Whelan J., Hohenberger P. (2014). Doxorubicin alone versus intensified doxorubicin plus ifosfamide for first-line treatment of advanced or metastatic soft-tissue sarcoma: A randomised controlled phase 3 trial. Lancet Oncol..

[56] van Houdt W.J., Raut C.P., Bonvalot S., Swallow C.J., Haas R., Gronchi A. (2019). New research strategies in retroperitoneal sarcoma. The case of TARPSWG, STRASS and RESAR: Making progress through collaboration. Curr. Opin. Oncol..

[57] Woll P.J., Reichardt P., Le Cesne A., Bonvalot S., Azzarelli A., Hoekstra H.J., Leahy M., Van Coevorden F., Verweij J., Hogendoorn P.C. (2012). Adjuvant chemotherapy with doxorubicin, ifosfamide, and lenograstim for resected soft-tissue sarcoma (EORTC 62931): A multicentre randomised controlled trial. Lancet Oncol..

[58] Gronchi A., De Paoli A., Dani C., Merlo D.F., Quagliuolo V., Grignani G., Bertola G., Navarria P., Sangalli C., Buonadonna A. (2014). Preoperative chemo-radiation therapy for localised retroperitoneal sarcoma: A phase I-II study from the Italian Sarcoma Group. Eur. J. Cancer.

[59] Fairweather M., Gonzalez R.J., Strauss D., Raut C.P. (2018). Current principles of surgery for retroperitoneal sarcomas. J. Surg. Oncol..

[60] Tzanis D., Bouhadiba T., Gaignard E., Bonvalot S. (2018). Major vascular resections in retroperitoneal sarcoma. J. Surg. Oncol..

[61] Wortmann M., Alldinger I., Böckler D., Ulrich A., Hyhlik-Dürr A. (2017). Vascular reconstruction after retroperitoneal and lower extremity sarcoma resection. Eur. J. Surg. Oncol..

[62] Rossi C.R., Deraco M., De Simone M., Mocellin S., Pilati P., Foletto M., Cavaliere F., Kusamura S., Gronchi A., Lise M. (2004). Hyperthermic intraperitoneal intraoperative chemotherapy after cytoreductive surgery for the treatment of abdominal sarcomatosis: Clinical outcome and prognostic factors in 60 consecutive patients. Cancer.

[63] Lim S.J., Cormier J.N., Feig B.W., Mansfield P.F., Benjamin R.S., Griffin J.R., Chase J.L., Pisters P.W., Pollock R.E., Hunt K.K. (2007). Toxicity and outcomes associated with surgical cytoreduction and hyperthermic intraperitoneal chemotherapy (HIPEC) for patients with sarcomatosis. Ann. Surg. Oncol..

[64] Bonvalot S., Cavalcanti A., Le Péchoux C., Terrier P., Vanel D., Blay J.Y., Le Cesne A., Elias D. (2005). Randomized trial of cytoreduction followed by intraperitoneal chemotherapy versus cytoreduction alone in patients with peritoneal sarcomatosis. Eur. J. Surg. Oncol..

[65] Kusamura S., Raspagliesi F., Baratti D., Gronchi A., Casali P., Deraco M. (2016). Uterine Sarcoma Treated by Cytoreductive Surgery and Intraperitoneal Hyperthermic Perfusion: A Feasiblity Study. J. Chemother..

[66] Hayes-Jordan A., Green H., Fitzgerald N., Xiao L., Anderson P. (2010). Novel treatment for desmoplastic small round cell tumor: Hyperthermic intraperitoneal perfusion. J. Pediatr. Surg..

[67] Hayes-Jordan A., Green H.L., Lin H., Owusu-Agyemang P., Fitzgerald N., Arunkumar R., Mejia R., Okhuysen-Cawley R., Mauricio R., Fournier K. (2014). Complete cytoreduction and HIPEC improves survival in desmoplastic small round cell tumor. Ann. Surg. Oncol..

[68] Palussière J., Marcet B., Descat E., Deschamps F., Rao P., Ravaud A., Brouste V., de Baère T. (2011). Lung tumors treated with percutaneous radiofrequency ablation: Computed tomography imaging follow-up. Cardiovasc. Interv. Radiol..

[69] Pawlik T.M., Vauthey J.N., Abdalla E.K., Pollock R.E., Ellis L.M., Curley S.A. (2006). Results of a single-center experience with resection and ablation for sarcoma metastatic to the liver. Arch. Surg..

[70] Blackmon S.H., Shah N., Roth J.A., Correa A.M., Vaporciyan A.A., Rice D.C., Hofstetter W., Walsh G.L., Benjamin R., Pollock R. (2009). Resection of pulmonary and extrapulmonary sarcomatous metastases is associated with long-term survival. Ann. Thorac. Surg..

[71] Stephens E.H., Blackmon S.H., Correa A.M., Roth J.A., Rice D.C., Hofstetter W., Benjamin R., Mehran R., Swisher S.G., Walsh G.L. (2011). Progression after chemotherapy is a novel predictor of poor outcomes after pulmonary metastasectomy in sarcoma patients. J. Am. Coll. Surg..

[72] Maluccio M.A., Covey A.M., Schubert J., Brody L.A., Sofocleous C.T., Getrajdman G.I., DeMatteo R., Brown K.T. (2006). Treatment of metastatic sarcoma to the liver with bland embolization. Cancer.

[73] Chapiro J., Duran R., Lin M., Mungo B., Schlachter T., Schernthaner R., Gorodetski B., Wang Z., Geschwind J.F. (2015). Transarterial chemoembolization in soft-tissue sarcoma metastases to the liver—the use of imaging biomarkers as predictors of patient survival. Eur. J. Radiol..

[74] Weiser M.R., Downey R.J., Leung D.H., Brennan M.F. (2000). Repeat resection of pulmonary metastases in patients with soft-tissue sarcoma. J. Am. Coll. Surg..

[75] Burt B.M., Ocejo S., Mery C.M., Dasilva M., Bueno R., Sugarbaker D.J., Jaklitsch M.T. (2011). Repeated and aggressive pulmonary resections for leiomyosarcoma metastases extends survival. Ann. Thorac. Surg..

[76] Cardona K., Williams R., Movva S. (2013). Multimodality therapy for advanced or metastatic sarcoma. Curr. Probl. Cancer.

[77] Wagner M.J., Amodu L.I., Duh M.S., Korves C., Solleza F., Manson S.C., Diaz J., Neary M.P., Demetri G.D. (2015). A retrospective chart review of drug treatment patterns and clinical outcomes among patients with metastatic or recurrent soft tissue sarcoma refractory to one or more prior chemotherapy treatments. BMC Cancer.

[78] Leahy M., Garcia Del Muro X., Reichardt P., Judson I., Staddon A., Verweij J., Baffoe-Bonnie A., Jönsson L., Musayev A., Justo N. (2012). Chemotherapy treatment patterns and clinical outcomes in patients with metastatic soft tissue sarcoma. The SArcoma treatment and Burden of Illness in North America and Europe (SABINE) study. Ann. Oncol..

[79] Ryan C.W., Merimsky O., Agulnik M., Blay J.Y., Schuetze S.M., Van Tine B.A., Jones R.L., Elias A.D., Choy E., Alcindor T. (2016). PICASSO III: A Phase III, Placebo-Controlled Study of Doxorubicin with or Without Palifosfamide in Patients with Metastatic Soft Tissue Sarcoma. J. Clin. Oncol..

[80] Judson I., Radford J.A., Harris M., Blay J.Y., van Hoesel Q., le Cesne A., van Oosterom A.T., Clemons M.J., Kamby C., Hermans C. (2001). Randomised phase II trial of pegylated liposomal doxorubicin (DOXIL/CAELYX) versus doxorubicin in the treatment of advanced or metastatic soft tissue sarcoma: A study by the EORTC Soft Tissue and Bone Sarcoma Group. Eur. J. Cancer.

[81] Sleijfer S., Ouali M., van Glabbeke M., Krarup-Hansen A., Rodenhuis S., Le Cesne A., Hogendoorn P.C., Verweij J., Blay J.Y. (2010). Prognostic and predictive factors for outcome to first-line ifosfamide-containing chemotherapy for adult patients with advanced soft tissue sarcomas: An exploratory, retrospective analysis on large series from the European Organization for Research and Treatment of Cancer-Soft Tissue and Bone Sarcoma Group (EORTC-STBSG). Eur. J. Cancer.

[82] Stacchiotti S., Libertini M., Negri T., Palassini E., Gronchi A., Fatigoni S., Poletti P., Vincenzi B., Dei Tos A.P., Mariani L. (2013). Response to chemotherapy of solitary fibrous tumour: A retrospective study. Eur. J. Cancer.

[83] Tap W.D., Wagner A.J., Schöffski P., Martin-Broto J., Krarup-Hansen A., Ganjoo K.N., Yen C.C., Abdul Razak A.R., Spira A., Kawai A. (2020). Effect of Doxorubicin Plus Olaratumab vs Doxorubicin Plus Placebo on Survival in Patients with Advanced Soft Tissue Sarcomas: The ANNOUNCE Randomized Clinical Trial. JAMA.

[84] D’Ambrosio L., Touati N., Blay J.Y., Grignani G., Flippot R., Czarnecka A.M., Piperno-Neumann S., Martin-Broto J., Sanfilippo R., Katz D. (2020). Doxorubicin plus dacarbazine, doxorubicin plus ifosfamide, or doxorubicin alone as a first-line treatment for advanced leiomyosarcoma: A propensity score matching analysis from the European Organization for Research and Treatment of Cancer Soft Tissue and Bone Sarcoma Group. Cancer.

[85] Stacchiotti S., Tortoreto M., Bozzi F., Tamborini E., Morosi C., Messina A., Libertini M., Palassini E., Cominetti D., Negri T. (2013). Dacarbazine in solitary fibrous tumor: A case series analysis and preclinical evidence vis-a-vis temozolomide and antiangiogenics. Clin. Cancer Res..

[86] Martin-Liberal J., Alam S., Constantinidou A., Fisher C., Khabra K., Messiou C., Olmos D., Mitchell S., Al-Muderis O., Miah A. (2013). Clinical activity and tolerability of a 14-day infusional Ifosfamide schedule in soft-tissue sarcoma. Sarcoma.

[87] Lorigan P., Verweij J., Papai Z., Rodenhuis S., Le Cesne A., Leahy M.G., Radford J.A., Van Glabbeke M.M., Kirkpatrick A., Hogendoorn P.C. (2007). Phase III trial of two investigational schedules of ifosfamide compared with standard-dose doxorubicin in advanced or metastatic soft tissue sarcoma: A European Organisation for Research and Treatment of Cancer Soft Tissue and Bone Sarcoma Group Study. J. Clin. Oncol..

[88] Demetri G.D., von Mehren M., Jones R.L., Hensley M.L., Schuetze S.M., Staddon A., Milhem M., Elias A., Ganjoo K., Tawbi H. (2016). Efficacy and Safety of Trabectedin or Dacarbazine for Metastatic Liposarcoma or Leiomyosarcoma After Failure of Conventional Chemotherapy: Results of a Phase III Randomized Multicenter Clinical Trial. J. Clin. Oncol..

[89] Le Cesne A., Blay J.Y., Judson I., Van Oosterom A., Verweij J., Radford J., Lorigan P., Rodenhuis S., Ray-Coquard I., Bonvalot S. (2005). Phase II study of ET-743 in advanced soft tissue sarcomas: A European Organisation for the Research and Treatment of Cancer (EORTC) soft tissue and bone sarcoma group trial. J. Clin. Oncol..

[90] Demetri G.D., Schöffski P., Grignani G., Blay J.Y., Maki R.G., Van Tine B.A., Alcindor T., Jones R.L., D’Adamo D.R., Guo M. (2017). Activity of Eribulin in Patients with Advanced Liposarcoma Demonstrated in a Subgroup Analysis From a Randomized Phase III Study of Eribulin Versus Dacarbazine. J. Clin. Oncol..

[91] Schöffski P., Chawla S., Maki R.G., Italiano A., Gelderblom H., Choy E., Grignani G., Camargo V., Bauer S., Rha S.Y. (2016). Eribulin versus dacarbazine in previously treated patients with advanced liposarcoma or leiomyosarcoma: A randomised, open-label, multicentre, phase 3 trial. Lancet.

[92] Seddon B., Scurr M., Jones R.L., Wood Z., Propert-Lewis C., Fisher C., Flanagan A., Sunkersing J., A’Hern R., Whelan J. (2015). A phase II trial to assess the activity of gemcitabine and docetaxel as first line chemotherapy treatment in patients with unresectable leiomyosarcoma. Clin. Sarcoma Res..

[93] Pautier P., Floquet A., Penel N., Piperno-Neumann S., Isambert N., Rey A., Bompas E., Cioffi A., Delcambre C., Cupissol D. (2012). Randomized multicenter and stratified phase II study of gemcitabine alone versus gemcitabine and docetaxel in patients with metastatic or relapsed leiomyosarcomas: A Federation Nationale des Centres de Lutte Contre le Cancer (FNCLCC) French Sarcoma Group Study (TAXOGEM study). Oncologist.

[94] García-Del-Muro X., López-Pousa A., Maurel J., Martín J., Martínez-Trufero J., Casado A., Gómez-España A., Fra J., Cruz J., Poveda A. (2011). Randomized phase II study comparing gemcitabine plus dacarbazine versus dacarbazine alone in patients with previously treated soft tissue sarcoma: A Spanish Group for Research on Sarcomas study. J. Clin. Oncol..

[95] Maki R.G., Wathen J.K., Patel S.R., Priebat D.A., Okuno S.H., Samuels B., Fanucchi M., Harmon D.C., Schuetze S.M., Reinke D. (2007). Randomized phase II study of gemcitabine and docetaxel compared with gemcitabine alone in patients with metastatic soft tissue sarcomas: Results of sarcoma alliance for research through collaboration study 002 [corrected]. J. Clin. Oncol..

[96] Patel S.R., Gandhi V., Jenkins J., Papadopolous N., Burgess M.A., Plager C., Plunkett W., Benjamin R.S. (2001). Phase II clinical investigation of gemcitabine in advanced soft tissue sarcomas and window evaluation of dose rate on gemcitabine triphosphate accumulation. J. Clin. Oncol..

[97] van der Graaf W.T., Blay J.Y., Chawla S.P., Kim D.W., Bui-Nguyen B., Casali P.G., Schöffski P., Aglietta M., Staddon A.P., Beppu Y. (2012). Pazopanib for metastatic soft-tissue sarcoma (PALETTE): A randomised, double-blind, placebo-controlled phase 3 trial. Lancet.

[98] Frezza A.M., Jones R.L., Lo Vullo S., Asano N., Lucibello F., Ben-Ami E., Ratan R., Teterycz P., Boye K., Brahmi M. (2018). Anthracycline, Gemcitabine, and Pazopanib in Epithelioid Sarcoma: A Multi-institutional Case Series. JAMA Oncol..

[99] Wagner A.J., Malinowska-Kolodziej I., Morgan J.A., Qin W., Fletcher C.D., Vena N., Ligon A.H., Antonescu C.R., Ramaiya N.H., Demetri G.D. (2010). Clinical activity of mTOR inhibition with sirolimus in malignant perivascular epithelioid cell tumors: Targeting the pathogenic activation of mTORC1 in tumors. J. Clin. Oncol..

[100] van Dalen T., Hoekstra H.J., van Geel A.N., van Coevorden F., Albus-Lutter C., Slootweg P.J., Hennipman A. (2001). Locoregional recurrence of retroperitoneal soft tissue sarcoma: Second chance of cure for selected patients. Eur. J. Surg. Oncol..

[101] Neuhaus S.J., Barry P., Clark M.A., Hayes A.J., Fisher C., Thomas J.M. (2005). Surgical management of primary and recurrent retroperitoneal liposarcoma. Br. J. Surg..

[102] Lochan R., French J.J., Manas D.M. (2011). Surgery for retroperitoneal soft tissue sarcomas: Aggressive re-resection of recurrent disease is possible. Ann. R. Coll. Surg. Engl..

[103] Anaya D.A., Lahat G., Liu J., Xing Y., Cormier J.N., Pisters P.W., Lev D.C., Pollock R.E. (2009). Multifocality in retroperitoneal sarcoma: A prognostic factor critical to surgical decision-making. Ann. Surg..

[104] Park J.O., Qin L.X., Prete F.P., Antonescu C., Brennan M.F., Singer S. (2009). Predicting outcome by growth rate of locally recurrent retroperitoneal liposarcoma: The one centimeter per month rule. Ann. Surg..

[105] Sanfilippo R., Bertulli R., Marrari A., Fumagalli E., Pilotti S., Morosi C., Messina A., Dei Tos A.P., Gronchi A., Casali P.G. (2014). High-dose continuous-infusion ifosfamide in advanced well-differentiated/dedifferentiated liposarcoma. Clin. Sarcoma Res..

[106] Toulmonde M., Le Cesne A., Mendiboure J., Blay J.Y., Piperno-Neumann S., Chevreau C., Delcambre C., Penel N., Terrier P., Ranchère-Vince D. (2014). Long-term recurrence of soft tissue sarcomas: Prognostic factors and implications for prolonged follow-up. Cancer.

